# Characterization and Growth Kinetics of Borides Layers on Near-Alpha Titanium Alloys

**DOI:** 10.3390/ma17194815

**Published:** 2024-09-30

**Authors:** Rongxun Piao, Wensong Wang, Biao Hu, Haixia Hu

**Affiliations:** 1 School of Mechanical and Electrical Engineering, Anhui University of Science and Technology, Huainan 232001, China; 15225068736@163.com (W.W.); huhx2006@163.com (H.H.); 2 Anhui Intelligent Mine Technology and Equipment Engineering Research Center, Huainan 232001, China; 3 School of Materials Science and Engineering, Anhui University of Science and Technology, Huainan 232001, China; hubiao05047071@163.com

**Keywords:** near-α type titanium alloy, pack boriding method, growth kinetics of boride layers, friction coefficient

## Abstract

Pack boriding with CeO_2_ was performed on the powder metallurgical (PM) near-α type titanium alloy at a temperature of 1273–1373 K for 5–15 h followed by air cooling. The microstructure analysis showed that the boride layer on the surface of the alloy was mainly composed of a monolithic TiB_2_ outer layer, inner whisker TiB and sub-micron sized flake-like TiB layer. The growth kinetics of the TiB_2_ and TiB layers obeyed the parabolic diffusion model. The diffusion coefficient of boron in the boride layers obtained in the present study was well within the ranges reported in the literature. The activation energies of boron in the TiB_2_ and TiB layers during the pack boriding were estimated to be 166.4 kJ/mol and 122.8 kJ/mol, respectively. Friction tests showed that alloys borided at moderate temperatures and times had lower friction coefficients, which may have been due to the fine grain strengthening effect of TiB whiskers. The alloy borided at 1273 K for 10 h had a minimum friction coefficient of 0.73.

## 1. Introduction

Near-alpha titanium alloys containing major alloying elements of Al–Zr(–Sn) are widely used in aircraft engine components, aircraft partitions frames and ribbed panels due to their excellent high-temperature creep resistance, good thermal stability, high strength to weight ratio and good corrosion resistance [1,2]. However, like most titanium alloys, it has extremely poor tribological properties by low surface hardness and poor wear resistance, which limits its wider application in wear-resistant parts such as certain mechanical structures, transmission gears, bearings and brakes [3,4,5,6].

Boriding, as a thermochemical surface treatment method, is an effective way to improve the wear resistance of titanium and its alloys by forming a harder boride layer on the surface [7,8,9]. Boriding treatment can be conducted by various methods, including pack boriding [7,10], plasma assisted boriding [11], paste boriding [10], ion boriding [11], laser boriding [12], etc. Among them, pack boriding not only meets the requirements of improving hardness and wear resistance, but also has the advantages of a simple process and equipment, convenient operation and simplified workpiece cleaning [13,14].

During the boriding process, the diffusion of boron into the substrate determines the growth rate of hard dual TiB_2_ and TiB layers [15], depending on processing parameters such as the boriding temperature and boriding time. By developing a growth kinetic model, the nature of growth of titanium borides on the surface of Ti alloy can be explored. Previously, a few studies of growth kinetic model have been reported in the pack boriding process. Li et al. [16] studied the growth kinetics of titanium boride layers on the surface of Ti–6Al–4V (TC4) using the diffusion model $d^{2}=Dt$, where the square of the boride layer thickness was directly proportional to the boriding time. By analyzing the growth kinetics of the total thickness of the boride layer, the diffusivities of boron at 1273–1373 K was determined as the order of magnitude 10^−15^ m^2^/s, and the average diffusion activation energy of boron atom in Ti-6Al-4V alloy was 65.2 kJ/mol. Li et al. [13] used the diffusion model $d=kt^{0.5}$ to study the growth kinetics of boride layer thickness in Ti–5Mo–5V–8Cr–3Al (TB2) alloy. This model was based on solving the mass balance equations at two (TiB_2_/TiB and TiB/Ti) interfaces by considering the parabolic growth constants. By comparing the two models of $d=kt^{0.5}$ and $d^{2}=Dt$, they concluded that the diffusion model of $d=kt^{0.5}$ was found to predict the thickness of boride layer more accurately. Similarly, Liu et al. [17] suggested that the growth kinetics of boride layers in Ti−5Mo−5V−8Cr−3Al (TB2) alloy during pack boriding with REs catalyst conformed to the parabolic diffusion model of $d=kt^{0.5}$ instead of $d^{2}=Dt$. Sarma et al. [18] studied the kinetics of growth of boride layers during pack boriding of Cp-Ti, developed the parabolic model by considering error function solution and concluded that the growth of TiB_2_ as well as that of the total (TiB_2_ + TiB) layer was obeyed the parabolic kinetics. Recently, Xing et al. [19] investigated the growth kinetics of boride layers of Ti−5Al−2.5Sn (TA7) alloy using both models of $d=kt^{0.5}$ and $d^{2}=Dt$ and suggested that the diffusion model of $d^{2}=Dt$ was more accurate in predicting the thickness of boride layer compared to the parabolic model. Ouyang et al. [3] predicted the growth kinetics of boride layers of near-α Ti−6Al−2Zr−1Mo−1V (TA15) alloy by establishing a parabolic diffusion model $d=kt^{0.5}$, and their model predictions presented satisfactory consistency with the experimental data. At present, there are two main diffusion models used in investigating the growth kinetics of boride layers of different Ti alloys. Although these models’ predictions show reasonable agreement to experimental data, limited studies have been performed on the growth kinetics of boride layers during pack boriding of near-α Ti alloys.

This work aimed to investigate the growth kinetics of boride layers in the near-α Ti alloys during the pack boriding process. For this purpose, the characterization of the boride layers on the surface of the alloy was studied. The growth kinetics model was established based on the parabolic growth law together with the mass balance equation, and a series of comparisons were made between the estimated diffusion coefficients and activation energy data and the reported data. Additionally, the wear property of near-α Ti alloys after pack boriding process was also discussed.

## 2. Experimental Procedure

**Figure 1:**
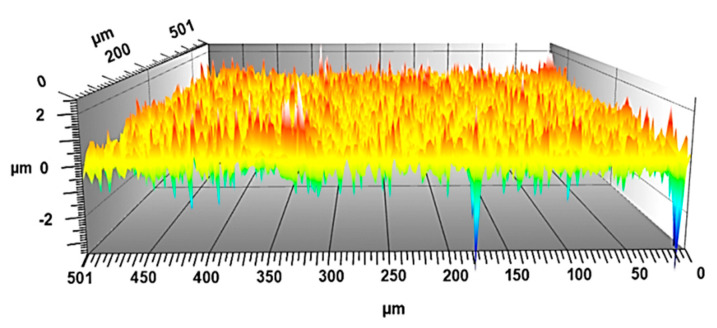
Three-dimensional topography of the polished surface of the PM Ti−5.1Al−2.0Sn−3.7Zr−0.3Mo−0.3Si alloy.

The alloy used in this study were prepared by powder metallurgy using vacuum sintering and a hot isostatic pressing processes [20]. The nominal chemical composition of the alloy is given in Table 1. The alloy was cut into the samples with sizes of 15 mm × 10 mm × 3 mm by a wire-cutting machine. The samples were ground using emery paper and polished with diamond polishing paste to an average roughness of Ra ≤ 0.4 μm (Figure 1), and then ultrasonic cleaning was carried out in acetone.

Before the solid-state boriding heat treatment, boriding agent was made by mixing 94wt% B_4_C (as the boron source) and 6wt% CeO_2_ (as the catalyst) with particle size ≤ 200 mesh. Then, the samples were sealed in a corundum crucible (Changsha Dongyan Advanced Materials Co., LTD., Changsha, China) with a lid by tightly packing the boronizing agent powder around the sample. The boriding temperature was chosen in the range of 1273–1373 K near the α/β transition temperature (T_β_ = 1293 K [20]), which is generally considered to have good boriding effects [18]. Thus, the rare-earth-catalyzed boriding of the Ti−5.1Al−2.0Sn−3.7Zr−0.3Mo−0.3Si alloy was performed at 1273–1373 K for 5–15 h. After reaching the target temperature and holding time for boronizing, the corundum crucible containing the boronized sample was taken out from the furnace and cooled in the air.

After the boriding heat treatment, the cross section of the boronized sample was ground and polished, cleaned with acetone and then etched in Kroll’s reagent (1 mL HF, 5 mL HNO_3_ and 100 mL water). The microstructure of the boride layer on the cross section of the sample was observed by the FlexSEM1000 scanning electron microscope (SEM, TESCAN-MIRA 4, Tescan Co., LTD., Shanghai, China) with an energy dispersive spectrometer (EDS) and DMM490C optical microscope (OM, Caikang Optical Instrument Co., LTD., Shanghai, China). The thickness of boriding layers was determined by taking the average of at least 5–10 measurements on each sample [3], and the details of the measurements can be found in the Supplementary Materials. The microhardness measurement was carried out on the HV-1000Z microhardness tester (Deka precision meter Co., LTD., Shenzhen, China) under the conditions of a test force of 10 gf and a holding time of 15 s. Hardness testing was performed from the surface of the boriding layer toward the substrate at an interval of 2–3 μm at the boriding layer and 5 μm at the substrate. The phase analysis of the boronized surface of the sample was carried out by X-ray diffraction (XRD). The dry friction test was conducted at room temperature using the ball-on-plate linear reciprocating GF-I tribometer (Zhongke Kaihua Technology, Lanzhou, China). During the test, an Al_2_O_3_ ball with a diameter of Ø10 mm was selected as the counter material, and the reciprocating sliding test was conducted under normal load of 5N, constant sliding speed of 0.06 m/s, sliding length of 5 mm and duration of 10 min. The measured value at 10 min was taken as the final friction coefficient of the alloy.

## 3. Results

### 3.1. Characterization of Titanium Boride Layers

**Figure 2:**
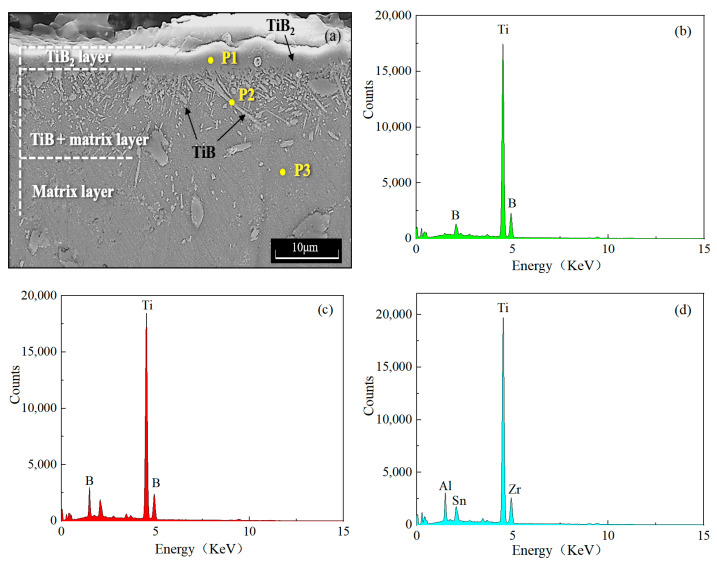
SEM micrograph of the Ti−5.1Al−2.0Sn−3.7Zr−0.3Mo−0.3Si alloy borided at 1323 K for 10 h (**a**), and EDS point analysis at P1 (**b**), P2 (**c**) and P3 (**d**) in (**a**).

Figure 2a shows the typical characteristics of the surface boride layer on the cross-section of the Ti−5.1Al−2.0Sn−3.7Zr−0.3Mo−0.3Si alloy. It can be seen in Figure 2a that the boride layer exhibited a structure of a monolithic TiB_2_ outer layer and an inner layer of whisker TiB penetrating into the substrate. Similar morphologies have been confirmed in various dual-phase titanium alloys [3,4]. The different morphologies between TiB_2_ and TiB layers may be attributed to the different preferential growth directions of TiB_2_ and TiB crystals [21]. It is generally known that the hexagonal TiB_2_ has a preferential growth direction on *ab* plane perpendicular to the [0001] direction, selectively forming a dense structure [21,22], while TiB has a preferential growth direction of [010], and there are some specific orientation relationships between TiB and titanium substrates, resulting in needle-like or whisker-like morphology along the [010] direction [3]. In both the TiB_2_ and TiB layers, no obvious microcracks or holes were observed, showing a good bonding structure between the boride layer and the substrate. The density measurements indicate that the density of the alloy increased from the original 4.5 g/cm^3^ to an average value of 4.73 g/cm^3^ after the boriding process, confirming the densification of the alloy. It is worth noting that a lot of short flaky or irregularly shaped TiB with sub-micron sizes (<0.7 μm) were also observed in the TiB + matrix layer. These sub-micron-sized TiB may have been formed by the reaction (Ti + [B] = TiB) between active [B] diffused from boron source through the outer layer of TiB_2_ and Ti from the alloy substrate. When the thickness of the TiB_2_ layer reached a certain value, it hindered the diffusion of active [B] atoms into the alloy substrate, thereby limiting the growth of the boride layer and forming short flaky morphology [22,23,24]. Another reason may be the presence of micropores in the alloy during powder metallurgy preparation. As preliminary density measurements, the density of the alloy used in present study was 4.5 g/cm, which was 97.1% of the theoretical density [20]. This different density indicates the presence of micropores in the matrix, which acted as anchor sites for boron diffusion, forming sub-micron-sized TiB phases. More research details need to be confirmed in future work.

**Figure 3:**
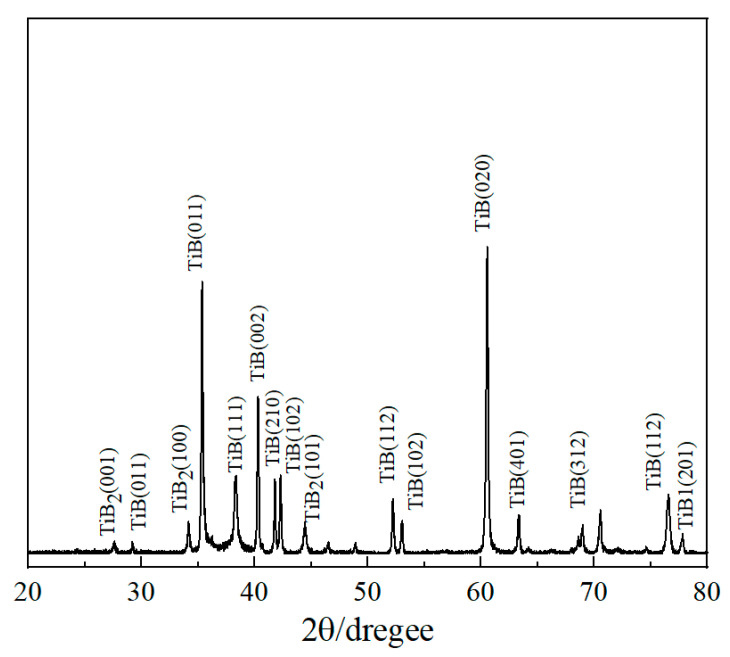
XRD pattern of the Ti−5.1Al−2.0Sn−3.7Zr−0.3Mo−0.3Si alloy borided at 1273 K for 10 h.

The EDS analyses show the presence of Ti and B in Figure 2b–c, as well as the main elements of the matrix in Figure 2d. The XRD measurements identified the existence of TiB2 and TiB phases as shown in Figure 3.

**Figure 4:**
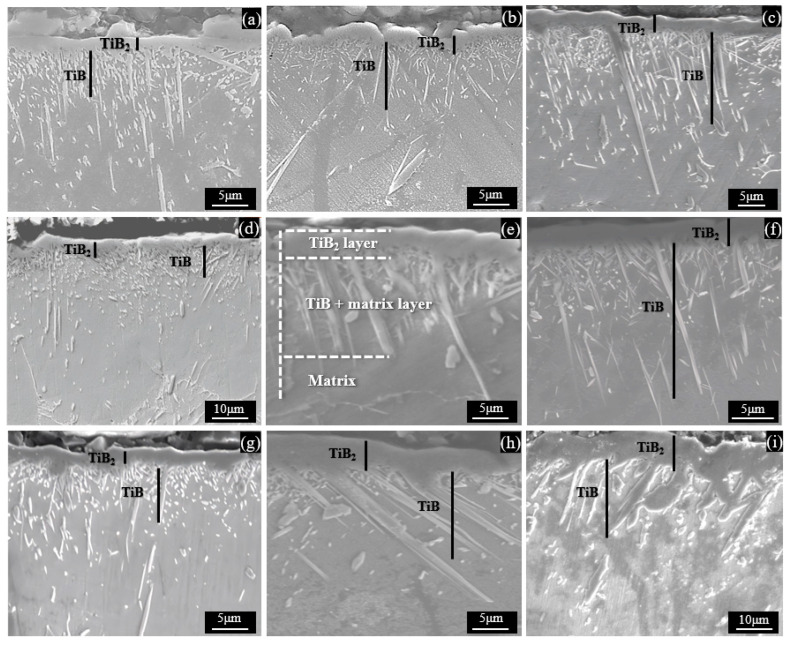
SEM micrographs of Ti−5.1Al−2.0Sn−3.7Zr−0.3Mo−0.3Si alloy borided for different condition: (**a**–**c**) 1273 K for 5, 10, 15 h; (**d**–**f**) 1323 K for 5, 10, 15 h; and (**g**–**i**) 1373 K for 5, 10, 15 h.

Figure 4 shows the SEM microstructure of Ti−5.1Al−2.0Sn−3.7Zr−0.3Mo−0.3Si alloys borided at different conditions. It can be clearly seen that there were three different regions, namely the outer layer of single TiB_2_, the inner layer of whiskers or discontinuous flaky TiB penetrating into the substrate and the substrate. The average thicknesses of the TiB_2_, TiB and total TiB_2_ + TiB layers at different boriding temperature and time are summarized in Table 2. The monolithic outer layer of TiB_2_ had a thickness of mostly less than 7 μm in all temperature ranges, and it had only a small portion of the total boride layer thickness. The whisker TiB had a thickness ranging from 7 μm to 23 μm under the present boriding conditions, and it accounted for a high proportion of the total boride layer thickness. Moreover, it can be seen from Figure 4a–d,g that the morphology of the alloy was characterized by a large number of sub-micron (<0.7 μm)-sized short flaky TiB phases under the boriding conditions of a relatively lower temperature (1273 K) and shorter time (5 h). With the increase of the boriding temperature and time, the TiB_2_ boride layer increased, and the TiB whiskers also became longer and thicker, while the tiny flaky TiB phase no longer existed in large quantities as shown in Figure 4e,f,h,i. According to above observations, it can be known that as the temperature and duration increased, surface [B] atoms became more active in diffusing into the substrate, resulting in an increase in the concentration of B atoms in the alloy matrix and accelerating the growth of the boride layer by merging small flake-like TiB phases.

**Figure 5:**
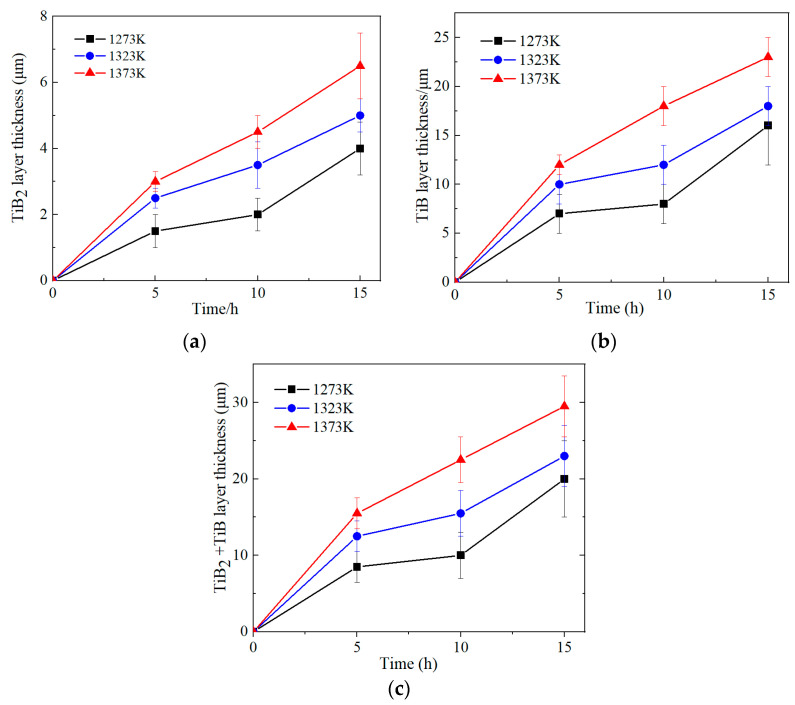
Changes in the boride layer thickness of the TiB_2_ layer (**a**), TiB layer (**b**) and TiB_2_ + TiB layer (**c**) under different conditions.

To clearly see the growth behavior of the boride layers, the variation relationships of boride layer thickness under different conditions are presented in Figure 5. The growth patterns of various boride layers were considered parabolic growth. As expected, the thickness of all boride layers increased with temperature and time. The outer layer of TiB_2_ showed a relatively slow growing tendency at the temperature of 1273–1323 K and heat treatment time below 10 h, followed by a relatively fast increase after 15 h. As the temperature rose to 1373 K, the thickness kinetic curve of the boride layers exhibited parabolic smooth growth. Since this temperature was well into the β-phase field of the alloy, the growth rate of boride layers was relatively higher than that at the temperature of 1273–1323 K. Similar growth behavior could be observed in TiB whisker, but the growth rate of this layer was much higher than that of TiB_2_. Due to the high proportion of TiB thickness out of the total layer, the growth kinetic curve of the total thickness of the boride layer was similar to that of the TiB layer.

### 3.2. Growth Kinetics of Boride Layers

**Figure 6:**
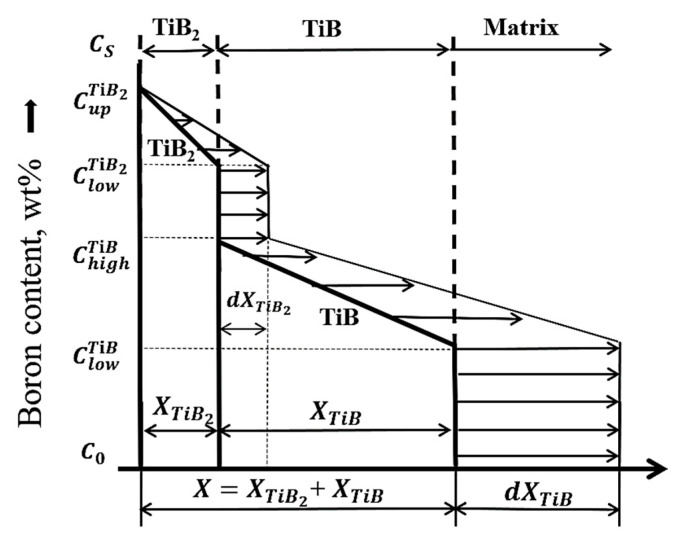
Schematic representation of B concentration profile across the boride layer. The *X* axis represents the depth from the surface (in μm). *C_S_* represents the B effective weight fraction in the boron source; is the upper and lower limits of B concentrations in TiB_2_, respectively; and denotes the lower and upper limits of B content in TiB; *C*_0_ is the solubility of B in the substrate, respectively.

It is known that diffusion of the B atom in Ti-based alloy is the main controlling step for the growth of TiB_2_ and TiB borides, so the growth of the boride layer is the result of B atoms diffusing into the substrate [16,25]. A diffusion-based parabolic model has widely been used to describe the growth kinetics of boride layers on titanium and its alloy [3,25,26,27]. Figure 6 shows a schematic diagram of the diffusion model considering the growth of the TiB_2_ and TiB whisker layers. In the model, the following assumptions were made [3,4]: (1) the growth of boride layer was controlled by the B diffusion, (2) the diffusion coefficient of B was concentration-independent, and (3) B diffusion in the Ti alloy matrix was negligible. Also, the growth of the TiB_2_ and TiB layers was considered as the displacements of the TiB_2_/TiB and TiB/Ti-substrate interfaces, and B concentration refers to the B concentration at the B/TiB_2_, TiB_2_/TiB and TiB/Ti-substate interfaces; thus, the boride thickness can be described as $\;d=kt^{0.5}$ using the parabolic growth constant and boriding time term. By combining mass balance equations [28,29] and introducing the correction factors of A and B, the B diffusion coefficient in TiB_2_ ($D_{B}^{{TiB}_{2}}$) and TiB ($D_{B}^{TiB}$) layers can be determined as follows [3]:

(1) $$D_{B}^{{TiB}_{2}}=A\frac{\left(w_{{TiB}_{2}}+w^{\prime }\right)k_{1}^{2}+w_{TiB}k_{1}k_{2}}{2(C_{Up}^{{TiB}_{2}}-C_{Low}^{{TiB}_{2}})}\;$$

(2) $$D_{B}^{TiB}=B\frac{{(k}_{2}-k_{1})\left(w_{TiB}k_{2}+w^{\prime }k_{1}\right)}{2(C_{Up}^{TiB}-C_{Low}^{TiB})}$$

with

(3) $$w_{{TiB}_{2}}=0.5\left(C_{Up}^{{TiB}_{2}}-C_{Low}^{{TiB}_{2}}\right)+(C_{Up}^{{TiB}_{2}}-C_{Low}^{TiB})\;$$

(4) $$w_{TiB}=0.5\left(C_{Up}^{TiB}-C_{Low}^{TiB}\right)+(C_{Low}^{TiB}-C_{0})$$

(5) $$w^{\prime }=0.5(C_{Up}^{TiB}-C_{Low}^{TiB})$$

Here, based on the model assumptions mentioned above, initial condition (for t = 0) of the boron solubility in the substate was set as C_0_ = 0, and boundary conditions (for t > 0) for the upper and lower limit of B concentration in boride layer were set as ${\;C}_{Up}^{{TiB}_{2}}\;$= 0.311, $C_{Low}^{{TiB}_{2}}=$0.301, $C_{Up}^{TiB}=0.185$ and $C_{Low}^{TiB}=0.18$ [3,4,10,18].

According to the parabolic growth model $d=kt^{0.5}$, the positions of the TiB_2_/TiB interface and TiB/Ti-substrate interface can be given as:

(6) $${x_{{TiB}_{2}}=k}_{1}t^{0.5}$$

(7) $$(x_{{TiB}_{2}}+x_{TiB}{)=k}_{2}t^{0.5}$$

Thus, the TiB layer thickness can be given as

(8) $$x_{TiB}=\left(x_{{TiB}_{2}}+x_{TiB}\right)-x_{{TiB}_{2}}{=(k}_{2}{-k}_{1})t^{0.5}$$

where $x_{{TiB}_{2}}$ and $x_{TiB}$ are the layers of TiB_2_ and TiB, and $k_{1}$ and $k_{2}$ are the parabolic growth constants at the TiB_2_/TiB and TiB/Ti interfaces, which can be determined by the linear fitting curve of $x_{{TiB}_{2}}-t^{0.5}$ and $x_{TiB}-t^{0.5}$.

**Figure 7:**
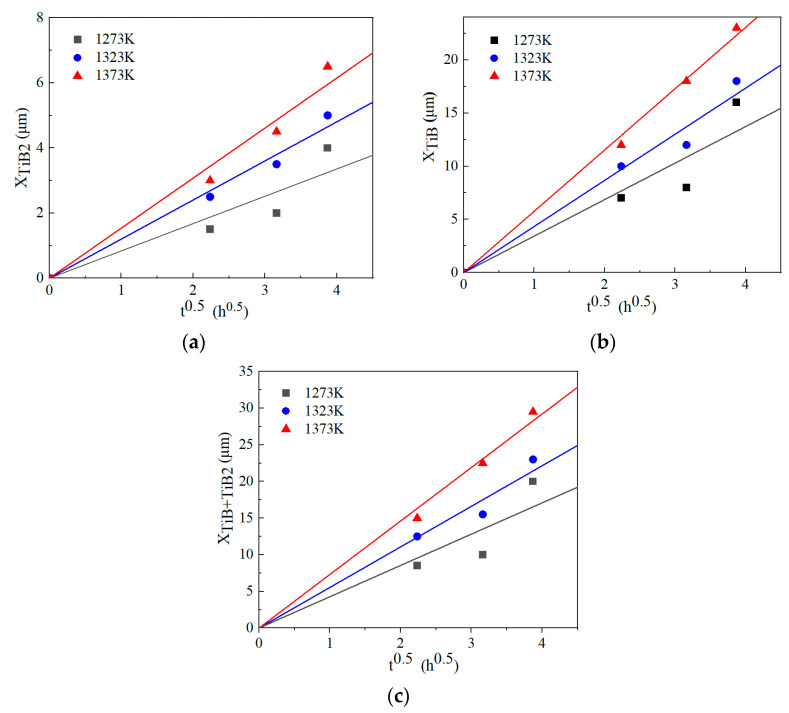
Plots of the boride layer thickness vs. square root of boriding time: (**a**) TiB_2_, (**b**) TiB and (**c**) TiB + TiB_2_.

Figure 7 shows the relationship between the boride thickness and square root of boriding time according to Equations (6)–(8). By analyzing the slope of linear fitting of curves in Figure 7, the parabolic growth constants at different boriding temperatures were determined and listed in Table 3. The parabolic growth constant showed an increasing tendency with time and temperature.

For better kinetic modeling and prediction, the correction factors of *A* and *B* for $D_{B}^{{TiB}_{2}}$ and $D_{B}^{TiB}$ were introduced based on the relationship of ${k=D}^{0.5}$ and determined as 0.0203 and 0.2508 through the following equations [3]:

(9) $$A=k_{1}^{2}/\frac{\left(w_{{TiB}_{2}}+w^{\prime }\right)k_{1}^{2}+w_{TiB}k_{1}k_{2}}{2(C_{up}^{{TiB}_{2}}-C_{low}^{{TiB}_{2}})}$$

(10) $$B=(k_{1}{-k}_{2}{)}^{2}/\frac{\left(w_{{TiB}_{2}}+w^{\prime }\right)k_{1}^{2}+w_{TiB}k_{1}k_{2}}{2(C_{up}^{{TiB}_{2}}-C_{low}^{{TiB}_{2}})}$$

As a result, the boron diffusion coefficients using parabolic constants in the boride layers can be determined based on Equations (1) and (2), and the calculation results at different temperatures are also listed in Table 3. Similar to the parabolic constants, the B diffusion coefficients in both TiB_2_ and TiB showed a gradual increasing tendency as the boriding temperature increased. As seen in Table 3, the diffusion coefficient of boron in TiB_2_ ranged from 2.33 × 10^−16^ to 7.16 × 10^−16^ m^2^·s^−1^, and the diffusion coefficient of boron in TiB was two orders of magnitude higher than that in TiB_2_, ranging from 2.33 × 10^−14^ to 5.45 × 10^−14^ m^2^ ·s^−1^. Table 4 lists the diffusion coefficients of boron in different borided Ti alloys calculated by the parabolic diffusion model of *d* = *kt*^0.5^. The diffusion coefficients of boron in the boride layers were typically of the order of 10^−16^ to 10^−13^, where the diffusion coefficient of boron in the *α*-type Ti alloy substrate was relatively lower than that in the *β*-type Ti alloy substrate. This is because B atoms have a lower diffusion energy barrier when diffusing in the *β*-type Ti alloy compared to the *α*-type Ti alloy [30]. Also, the diffusion coefficient of boron in TiB_2_ ($D_{B}^{{TiB}_{2}}$) was generally higher than that in TiB ($D_{B}^{TiB}$) layer. This was due to the dense structure of the TiB_2_ layer, which hinders the diffusion of the B atom [19]. The diffusion coefficient of boron in TiB_2_, $D_{B}^{{TiB}_{2}}$ obtained in the present study was well within the range of *α*-type Ti alloy substrates reported in the literature. On the other hand, the diffusion coefficient of boron in TiB, $D_{B}^{TiB}$ was within the range of Cp−Ti substrates but relatively higher than that in the *α*-type Ti−6Al−2Zr−1Mo−1V or Ti−5Al−2.5Sn alloy. This difference may have been caused by the different diffusion energy barriers influenced by the alloy elements. It has been recently reported that alloying elements dissolved in Ti substrate can segregate at the TiB/Ti interface, thus increasing the diffusion energy barrier for B atoms to further diffuse in multi-component Ti alloys compared to the clean (or without alloying) Ti matrix [22]. Another reason may have been the influence of boriding temperature. It can be seen in Table 4 that when boriding temperature was close to or higher than T_β_, the diffusion coefficient of boron usually showed a higher value than at a relatively lower temperature. The boriding temperature used in the present study was close to or higher than T*_β_* and higher than the temperature used in *α*-type Ti−6Al−2Zr−1Mo−1V or Ti−5Al−2.5Sn alloy, thereby showing a higher diffusion coefficient in the present study.

The temperature dependence of boron diffusion in both layers was analyzed by Arrhenius-type expression:

(11) $$D=D_{0}exp(-\frac{Q}{RT})$$

where Q is the diffusion activation energy (kJ/mol) of boron in a relevant boride layer, $D_{0}$ is the pre-exponential factor (m^2^/s), R is the gas constant (kJ/(mol·K)) and T is the absolute temperature (K).

**Figure 8:**
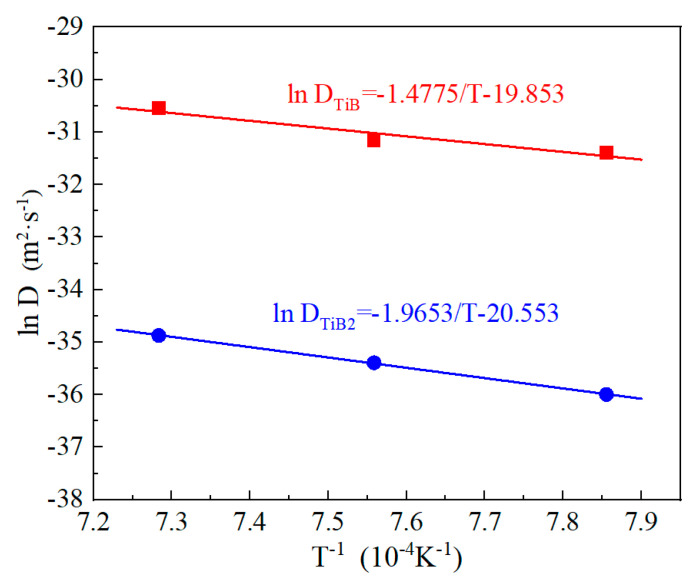
Linear relationship of lnD and 1/T.

By taking natural logarithm of Equation (11), the activation energy and exponential factor were determined from the slope and intercept of linear fitting between lnD and 1/T shown in Figure 8. Thus, the diffusion coefficients of boron in the TiB_2_ and TiB layers are given as follows:

(12) $$D_{B}^{{TiB}_{2}}=1.1856\times 10^{-9}exp(-\frac{166.4}{RT})$$

(13) $$D_{B}^{TiB}=2.3875\times 10^{-9}exp(-\frac{122.8}{RT})$$

The calculated activation energies of boron in the TiB_2_ layer and TiB layer were 166.4 kJ/mol and 122.8 kJ/mol, respectively. Due to the dense structural characteristics of the TiB_2_ layer, the diffusion of boron in this layer was relatively more difficult than in the TiB layer, so the boron diffusion activation energy in TiB_2_ layer was higher than that in the TiB layer. In addition, the growth of the TiB layer has a preferred growth direction in [010] [13], which can promote the growth of TiB whiskers. This means that the boron activation energy required for diffusion in the TiB layer is lower.

The reported values of the boron diffusion activation energies of borided Cp−Ti, Ti−6Al−2Zr−1Mo−1V, TA7, TC4 and TB2 titanium alloys are listed in Table 5 together with the values calculated in this study. As shown in table, the diffusion activation energies varied depending on the substrate, boriding method and temperature range. The activation energies of boron in the TiB_2_ and TiB layers obtained in present study were higher than that in Cp-Ti by plasma paste boriding. This was attributed to the diffusion barrier of alloy elements in the Ti−5Al−2Sn−3.7Zr−0.3Mo−0.3Si alloy hindering the diffusion of boron atom. Another reason was the use of plasma in Cp-Ti, which can generate extensive amounts of active boron and increase of activity of Ti and B [25]. In Ti−6Al−4V alloy, by plasma paste boriding, the boron activation energy ($Q_{B}^{{TiB}_{2}}\;$= 136.2 kJ/mol, $Q_{B}^{TiB\;}$= 63.8 kJ/mol) was lower than present study. This was also ascribed to the generation of active boron using plasma. Moreover, an increase of β-phase by the addition of V (acting as a β-phase stable element) may have been the reason for the promotion of boron diffusion in the Ti−6Al−4V alloy. Due to the same reason for high diffusion in the β-phase, the activation energy of boron diffusion in the β-type Ti−5Mo−5V−8Cr−3Al titanium alloy was lower than the results of present study. However, the diffusion activation energy (189.9 kJ/mol) of the boron in the TiB_2_ layer of CRTD (Cathodic Reduction and Thermal Diffusion)-based borided Cp-Ti, the diffusion activation energy of boron in the TiB_2_ and TiB layers of sigma fiber composite boronized Ti−6Al−4V and pack-borided Ti−6Al−2Zr−1Mo−1V, and the activation energy of boron in the TiB layer of the pack-borided Ti−5Al−2.5Sn alloy showed higher values than our results. This was due to the model used to estimate the boron activation energy is $d^{2}=Dt$, in which boron diffuses into the layers along the direction perpendicular to the interfaces, while the parabolic growth model $d=kt^{0.5}$ considers the growth of TiB_2_ and TiB layer as the displacements of TiB_2_/TiB and TiB/Ti-substrate interfaces [31]. Therefore, the boron activation energies estimated by the diffusion model $d^{2}=Dt$ in the literature [3,19,32,33] were higher than the present results given by the diffusion model $d=kt^{0.5}$.

**Figure 9:**
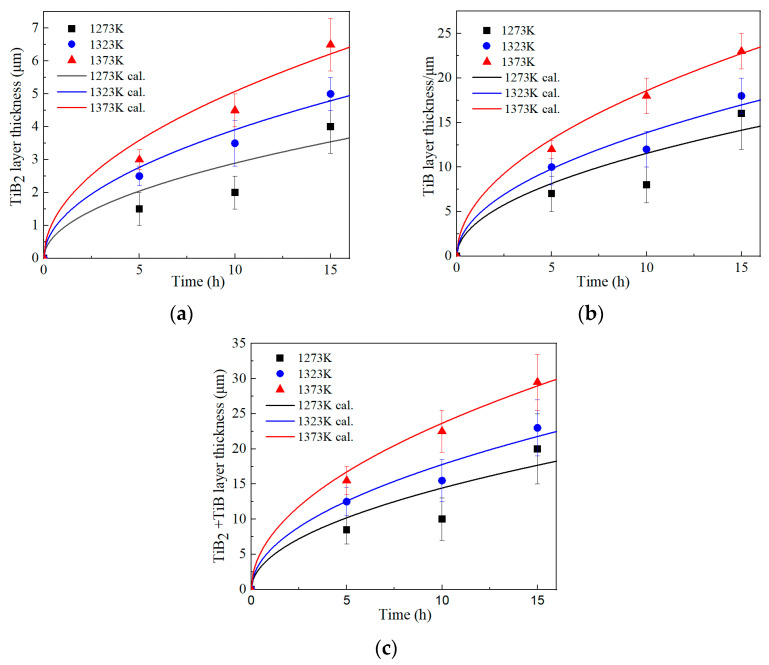
Comparison of the calculated and experimental values of boride layer thickness: (**a**) TiB_2_, (**b**) TiB and (**c**) TiB_2_ + TiB.

Figure 9 shows the boride growth kinetics curve calculated using the parabolic diffusion model based on Equations (6)–(8) together with the experimental data. The predicted thicknesses of the TiB_2_, TiB and total layer at relatively high temperatures of 1323 K and 1373 K were relatively consistent with experimental results, but the prediction at a low temperature of 1273 K slightly deviated from the experimental observations, especially at boriding times of 5 h and 10 h. This may be attributed to the fact that the TiB_2_ layer recedes by a complex mechanism involving enhanced anomalous diffusion in Ti near the β transition temperature [4]. Although some predictions may have some deviation, a reasonable agreement between predicted and experimental data was found in most cases within the error range, so the present model can be considered as a predictive model of the growth kinetics of the boriding layers for Ti−5Al−2Sn−3.7Zr−0.3Mo−0.3Si alloy.

### 3.3. Microhardenss and Wear Properties

**Figure 10:**
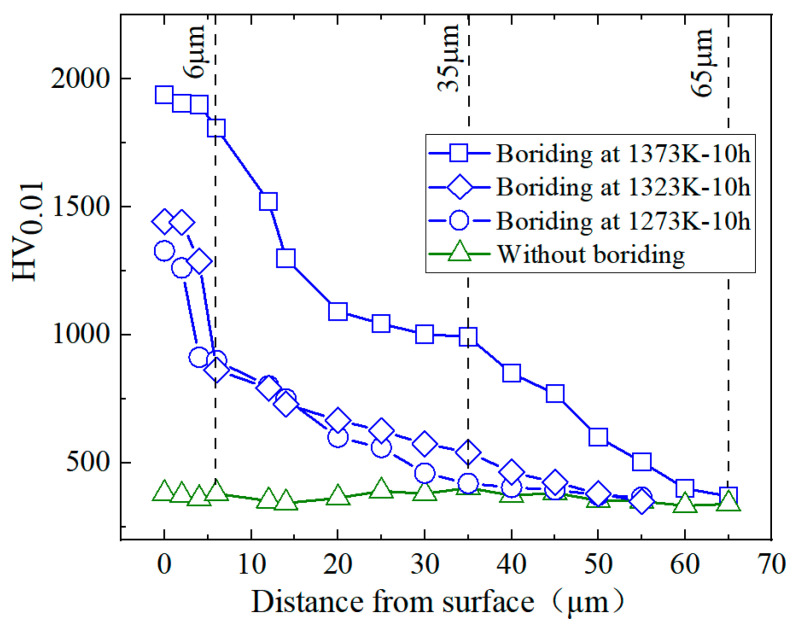
Variation of microhardenss with distance from the surface for the sample borided at 1373 K for 10 h and the as-sintered alloy.

The variation of microhardness with distance from the surface directly affected the wear resistance of the alloy. Figure 10 presents the microhardness from the surface to the interior for the as-sintered and the alloys pack borided at different temperatures for 10 h. It can be clearly seen that the microhardness of pack-borided alloys was higher than that of as-sintered alloys within a depth of 65 μm. Moreover, the maximum microhardness of the borided alloy was located at the top of the boriding layer, and it gradually decreased from the outside to the inside; on the other hand, no changes in microhardness were observed in the as-sintered alloy. As seen in the alloy borided 1373 K and 10 h, when the distance from surface was about 6μm, the microhardness changed from 1938 HV (19 GPa) to 1808 HV (17.7 GPa), approaching that of TiB_2_ (22 GPa) [34], thus considered as the outer layer of TiB_2_ within this distance range. When the distance was in the range from 6 μm to 35 μm, the microhardness decreased continuously from 1808 HV (17.7 GPa) to 993 HV (9.7 GPa), which was close to that of TiB (18.6 GPa) [35], thereby indicating that this range of distance is regarded as the TiB layer containing the matrix. As the distance increased further to the range from 35 μm to 65 μm, the microhardenss decreased linearly down to 370 HV (3.6 GPa). The microhardenss in this range was still higher than that of as-sintered alloy, although no boride phase was observed from the microsturue analysis. This implies that this area is still considered as TiB boride dispersed in the substrate. Similar results have been observed in TiB_2_ alloy [17]. When the distance exceeded 65 μm, the microhardness of the borided alloy was very close to the microhardness of the as-sintered alloy (350 HV or 3.4 GPa), so this range was considered as the matrix phase. As the boriding temperature decreased, the surface microhardness of the boride layers showed a decreasing tendency. The microhardness at surface of the alloy borided at relatively low temperature (1273 K and 1323 K) for 10 h showed a relatively lower value compared to that at 1373 K. This was attributed to the insufficient growth depth of the TiB_2_ layer at relatively low temperatures to resist surface pressure during hardness testing. Nevertheless, it clearly shows that the microhardness of the alloy surface was significantly improved after boriding compared to the alloy without boriding. These results imply that boride layers formed by pack boriding can greatly improve the surface hardness of the alloy, which is beneficial for improving the wear resistance of the alloy.

**Figure 11:**
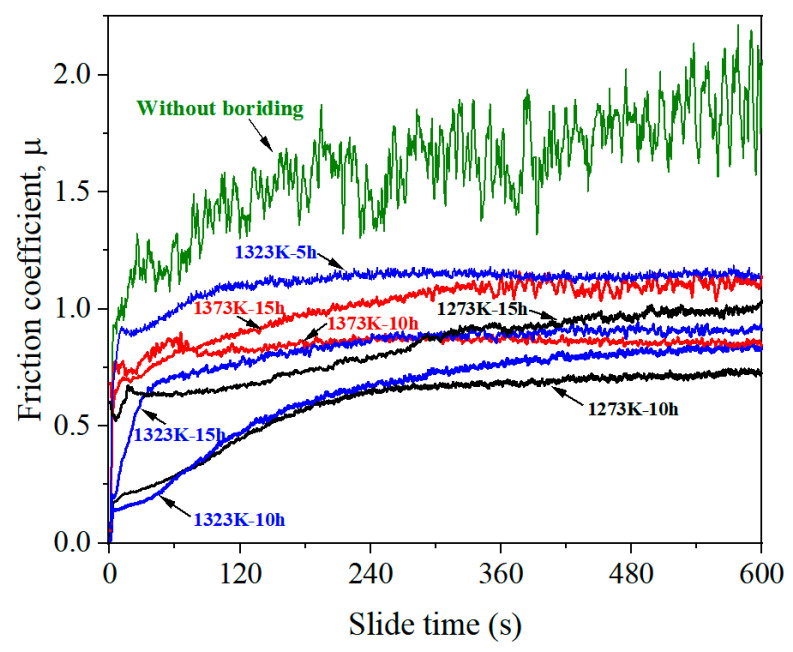
Friction coefficient of the sample borided at different conditions and the as-sintered alloy.

Figure 11 shows the friction coefficient of as-sintered and borided alloys under different reciprocating sliding test conditions. The friction test indicates that pack-borided alloy had a lower value of friction coefficient than that of as-sintered alloy during all test durations, due to the high surface hardness of the borided alloy. The friction coefficients observed at 600 s in this study ranged from 0.73 to 1.13, which was near half of the as–sintered alloy. Moreover, it can be seen that friction coefficient curve of the borided alloy showed a smooth trend, indicating a significant improvement in the surface quality of the alloy after pack boriding treatment. The friction coefficient of the alloys borided at 1323 K for 5 h showed a relatively high value of 1.13, showing relatively poor wear resistance. As treatment time increased to 10 h, the break-in period length increased, and the friction coefficient approached 0.83. As the processing time increased to 15 h, the friction coefficient did not decrease but slightly increased, reaching 0.92. Although the surface hardness increased with treatment time as shown in Figure 10, the friction coefficient showed a tendency to decrease first and then increase with time. Also, the friction coefficients of the alloy after 10 h of boronization at 1273 K and 1373 K were lower than the friction coefficient after 15 h of boronization at the corresponding temperature. In general, the growth of boride layer showed an increasing tendency of hardness simply due to the formation of harder boride phases, but it was not the only reason for the improvement of the wear property of the material. According to the surface uniaxial compression model [36,37], the wear volume of the boride layers is dependent not only on hardness but also on the size of TiB phase. When the size of TiB is small, a higher dislocation density is generated in the friction test near the TiB phase, leading to more improvement in surface tensile flow stress and further improvement in friction performance of the boride layers [37]. As discussed in Section 3.1, a lot of sub-micron-sized (<0.7 μm) TiB were observed in the PM-alloys borided at a relatively low temperature (1273 K and 1323 K) and short time durations (5–10 h), and the number of these tiny TiB whiskers decreased with increasing temperature and time. When the sub-micron-sized TiB whiskers were dominant, the wear resistance may have been mainly governed by fine-grain strengthening effect of the TiB whisker. As the boriding time increased from 10 h to 15 h at a fixed temperature, the amount of sub-micron-sized TiB decreased, while the TiB whiskers continued to grow longer and thicker, resulting in a higher friction coefficient over longer treatment times. When the boriding temperature was increased to 1373 K and the time was 15 h, the sub-micron-sized TiB basically disappeared, and the TiB whiskers grew abnormally, so the friction coefficient at this condition was quite high, showing poor wear performance. When the boriding temperature was 1273 K and the time was 10 h, the alloy had a relatively large amount of sub-micron-sized TiB with a certain length of TiB layers, demonstrating the best wear performance in this study.

## 4. Conclusions

The PM near-α titanium alloy was successfully borided by pack boriding with CeO_2_ at the temperature range of 1273–1373 K for 5–15 h. The boride layers on the surface of the alloy were composed of an outer TiB_2_ layer, a whisker-like TiB inner layer penetrating into the substrate and sub-micron TiB flakes distributed in the TiB + matrix layer. The thickness of both the TiB_2_ and TiB layers increased with the boriding temperature and time, in which the growth of TiB layer was more obvious. The parabolic diffusion model was used to predict the thickness of the boride layer, and the predicted results of the model were in good agreement with the experimental data within the experimental error range. The activation energies of boron in TiB_2_ and TiB layers during pack boriding were determined to be 166.4 kJ/mol and 122.8 kJ/mol, respectively. As the boriding temperature and time increased, the surface microhardness of the outer TiB_2_ layers showed an increasing tendency. The microhardness of the TiB_2_ outer layer of the alloy borided at 1373 K for 10 h was determined to be 1938 HV (19 GPa), which was almost five times higher than the as-sintered alloy. The friction coefficient of the borided alloy was in the range of 0.73~1.13, which was near half of the as-sintered alloy. The lowest friction coefficient of the borided alloy was observed after boriding at 1273 K for 10 h.

## Figures and Tables

**Table 1:** Chemical composition of near-alpha Ti alloy (mass%).

Al	Sn	Zr	Mo	Si	Ti
5.05	1.96	3.689	0.32	0.29	Bal.

**Table 2:** Averages thickness of the TiB_2_, TiB and total (TiB_2_ + TiB) layers at different treatment conditions.

T (K)	t (h)	d (μm)		
		TiB_2_	TiB	TiB_2_ + TiB
1273	5	1.5 ± 0.2	7 ± 4	8.5 ± 4
1273	10	2 ± 0.5	8 ± 2	10 ± 3
1273	15	4 ± 0.8	16 ± 4	20 ± 5
1323	5	2.5 ± 0.3	10 ± 4	12.5 ± 4
1323	10	3.5 ± 0.7	12 ± 5	15.3 ± 5
1323	15	5 ± 0.5	18 ± 7	23 ± 8
1373	5	3 ± 0.3	12 ± 2	15 ± 2
1373	10	4.5 ± 0.5	18 ± 2	22.5 ± 3
1373	15	6.5 ± 0.5	23 ± 5	29.8 ± 5

**Table 3:** Parabolic growth constants at TiB_2_/TiB and TiB/Ti-substrate interfaces, and diffusion coefficients at different boriding temperatures.

T (K)	$k_{1}$ (μm × s^−0.5^)	$k_{2}$ (μm × s^−0.5^)	$(k_{2}-k_{1}$) (μm × s^−0.5^)	$D_{B}^{{TiB}_{2}}$ (m^2^ × s^−1^)	$D_{B}^{TiB}$ (m^2^ × s^−1^)
1273	0.015	0.076	0.061	2.33 × 10^−16^	2.33 × 10^−14^
1323	0.021	0.094	0.073	4.25 × 10^−16^	2.92 × 10^−14^
1373	0.027	0.125	0.098	7.16 × 10^−16^	5.45 × 10^−14^

**Table 4:** The diffusion coefficients of boron in different borided Ti alloys calculated by the diffusion model of *d* = *kt*^0.5^.

Substrate	Boriding Methods	T*_β_*	T(K)	$D_{B}^{{TiB}_{2}}$ (m^2^ × s^−1^)	$D_{B}^{TiB}$ (m^2^ × s^−1^)
Ti5Al2Sn3.7Zr0.3Mo0.3Si (near-*α* type) [Present]	Pack boriding	1293 [20]	1273	2.33 × 10^−16^	2.33 × 10^−14^
			1323	4.25 × 10^−16^	2.92 × 10^−14^
			1373	7.16 × 10^−16^	5.45 × 10^−14^
Cp−Ti [18] (*α*-type)	Pack boriding	1183	1123	1.34 × 10^−16^	6.13 × 10^−15^
			1223	6.92 × 10^−16^	3.25 × 10^−14^
			1323	2.78 × 10^−15^	1.34 × 10^−13^
Ti6Al2Zr1Mo1V [3] (*α*-type)	Pack boriding	1253	1193	3.72 × 10^−17^	9.64 × 10^−16^
			1273	1.25 × 10^−16^	3.94 × 10^−15^
			1313	2.89 × 10^−16^	9.33 × 10^−15^
			1353	5.22 × 10^−16^	1.81 × 10^−14^
Ti5Al2.5Sn [19] (*α*-type, TA7)	Pack boriding	1298	1248	2.45 × 10^−14^	1.09 × 10^−15^
			1273	3.31 × 10^−14^	2.26 × 10^−15^
			1298	3.87 × 10^−14^	3.49 × 10^−15^
			1323	4.42 × 10^−14^	8.05 × 10^−15^
			1348	5.22 × 10^−14^	1.10 × 10^−14^
Ti6Al4V [10] (*α* + *β* type, TC4)	Plasma paste boriding	1183 [4]	973	5.08 × 10^−15^	1.90 × 10^−15^
			1023	7.25 × 10^−15^	4.32 × 10^−15^
			1073	10.18 × 10^−15^	9.13 × 10^−15^
Ti5Mo5V8Cr3Al (*β*-type,TB2) [13]	Pack boriding		1223	6.47 × 10^−15^	2.79 × 10^−14^
		-	1273	9.93 × 10^−15^	5.01 × 10^−14^
			1323	1.27 × 10^−14^	9.24 × 10^−14^
			1373	1.88 × 10^−14^	1.50 × 10^−13^
Ti5Mo5V8Cr3Al (*β*-type, TB2) [17]	Pack boriding	-	1223	4.20 × 10^−14^	7.35 × 10^−14^
			1273	8.42 × 10^−14^	1.06 × 10^−13^
			1323	1.39 × 10^−13^	1.55 × 10^−13^
			1373	2.05 × 10^−13^	1.93 × 10^−13^

**Table 5:** Diffusion activation energy of boron in the TiB_2_ and TiB layers.

Materials	Bording Method	Model	T (K)	$Q_{B}^{{TiB}_{2}}$ (kJ/mol)	$Q_{B}^{TiB}$ (kJ/mol)
Ti5Al2Sn3.7Zr0.3Mo0.3Si (near *α*-type) [present study]	Pack boriding	Parabolic model	1273–1373	166.4	122.8
Cp−Ti [15] (*α*-type)	Plasma paste boriding	Parabolic model	973–1073	137.6	55.2
Cp−Ti [32] (*α*-type)	CRTD based boriding	$d^{2}=Dt$	1173–1373	189.9	-
Ti6Al2Zr1Mo1V [3] (near *α*-type)	Pack boriding	$d^{2}=Dt$	1193–1353	223.1	246.9
Ti5Al2.5Sn [19] (*α*-type, TA7)	Pack boriding	$d^{2}=Dt$	1248–1348	94.8	221.2
Ti6Al4V [16] (*α* + *β* type, TC4)	Pack boriding	$d^{2}=Dt$	1273–1373	$65.2\;({=Q}_{B}^{{(TiB}_{2}+TiB)})$	
Ti6Al4V [10] (*α* + *β* type, TC4)	Plasma paste bording	Parabolic model	973–1073	136.2	63.8
Ti6Al4V [33] (*α* + *β* type, TC4)	Sigma fiber composites	$d^{2}=Dt$	1143–1243	187	190
Ti5Mo5V8Cr3Al [17] (*β*-type, TB2)	Pack boriding	Parabolic model	1223–1373	158.1	96.2

## Data Availability

The raw data supporting the conclusions of this article will be made available by the authors on request.

## Supplementary Materials

The following are available online at https://www.mdpi.com/article/10.3390/ma17194815/s1, Figure S1: Measurements of boride layer thickness of the samples borided under different conditions: (a) 1273 K for 10 h, (b) 1323 K for 10 h, and (c) 1373 K for 15 h.
