# Supplementary material for: Characterization and Growth Kinetics of Borides Layers on Near-Alpha Titanium Alloys
Source: Materials (Basel). 2024 Sep 30;17(19):4815. doi: 10.3390/ma17194815 (PMC11477631; doi:10.3390/ma17194815)
Supplement: Supplementary file 1 [file materials-17-04815-s001.zip › materials-3223360-supplementary.pdf]

Supplementary Information

Characterization and Growth Kinetics of Borides Layers on Near-Alpha Titanium Alloys

Rongxun Piao [1,2,*], Wensong Wang [1], Biao Hu [3] and Haixia Hu [1]

[1] School of Mechanical and Electrical Engineering, Anhui University of Science and Technology, Huainan 232001, China; 15225068736@163.com (W.W.); huhx2006@163.com (H.H.)
[2] Anhui Intelligent Mine Technology and Equipment Engineering Research Center, Huainan 232001, China
[3] School of Materials Science and Engineering, Anhui University of Science and Technology, Huainan 232001, China; hubiao05047071@163.com
* Correspondence: yhpark617@163.com

The thickness of the boriding layers is determined by taking the average of at least 5 to 10 measurements on each sample [3], and the details of the measurements can be found in Supplementary Information.

Firstly, the thickness of the TiB_2 layer and total (TiB_2+TiB) layer were determined by Equations (S1) and (S2):

$$d_{TiB_2} = \frac{\sum_i^n y_i}{n} \quad \text{(S1)}$$

$$d_{total} = \frac{\sum_i^n Y_i}{n} \quad \text{(S2)}$$

where $d_{TiB_2}$and d_{total} are average thicknesses of the TiB_2 and the total TiB_2+TiB layer, respectively; y_i and Y_i are the measured thicknesses of the TiB_2 and the total TiB_2+TiB layers, respectively; n is the number of measurements ($n \geq 5$).

Then, the thickness of TiB layer can be determined by Equation (S3) as follows:

$$d_{TiB} = d_{total} - d_{TiB_2} \quad \text{(S3)}$$

As the example, Figure S1 shows the thickness measurements of boride layers in the samples borided at 1273 K for 10 h, 1323 K/10 h, and 1373 K/15 h. Since the boride layers show features of protrusions, the thickness measurements should be conducted carefully. At least 5 to 10 measurements were made of each sample and averaged. By using Equations (S1) and (S2), the average thickness of TiB_2 and total TiB_2+TiB layers can be calculated. Considering the error range or deviation of measurement values, the final thickness of TiB_2 layers at 1273 K for 10 h, 1323 K/10 h, and 1373 K/15 h can be determined as 15 ± 0.2 μm, 3.5 ± 0.7 μm, 6.5 ± 0.5 μm, respectively, while the thickness of the total boride (TiB_2+TiB) is 8.5 ± 4.5 μm, 15.3 ± 5 μm, and 29.8 ± 5.2 μm, respectively. Then, according to Equation (S3), the thickness of TiB layers can be determined. All results are summarized in Table 1. Similar measurement method can be found in reference [3].

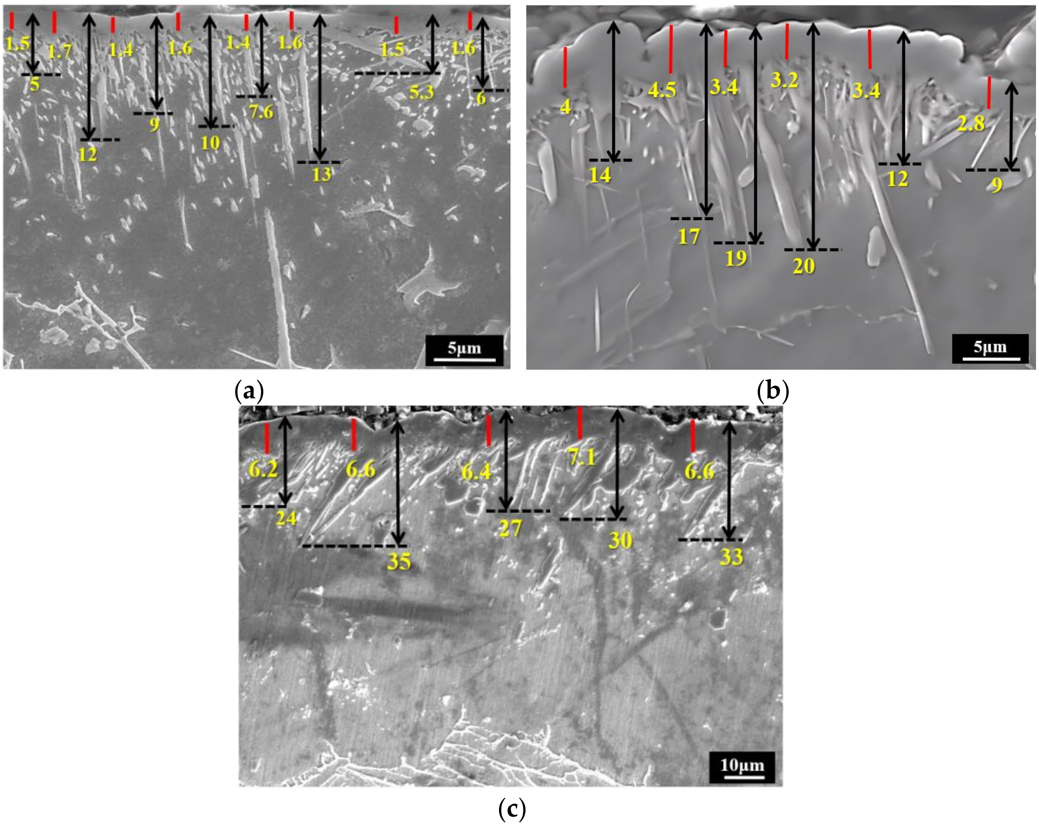

Figure S1. Measurements of boride layer thickness of the samples borided under different conditions: (**a**) 1273 K for 10 h, (**b**) 1323 K for 10 h , and (**c**) 1373 K for 15 h.
